# Chitosan Beads Incorporated with Essential Oil of *Thymus capitatus*: Stability Studies on Red *Tilapia* Fillets

**DOI:** 10.3390/biom9090458

**Published:** 2019-09-07

**Authors:** Mayra Alejandra Valencia Junca, Cesar Valencia, Edwin Flórez López, Johannes Delgado-Ospina, Paula A. Zapata, Moisés Solano, Carlos David Grande Tovar

**Affiliations:** 1 Grupo de Investigación en Química y Biotecnología QUIBIO, Universidad Santiago de Cali, Calle 5 No 62-00, Cali 760035, Colombia; aleja_valencia2201@hotmail.com (M.A.V.J.); edwin.florez00@usc.edu.co (E.F.L.); 2 SIMERQO Laboratorio de Polímeros, Departamento de Química, Universidad del Valle, Calle 13 No 100-00, Cali 760031, Colombia; cesar.valencia@correounivalle.edu.co; 3 Grupo de Investigación Biotecnología, Facultad de Ingeniería, Universidad de San Buenaventura Cali, Carrera 122 # 6-65, Cali 76001, Colombia; jdelgado1@usbcali.edu.co; 4 Grupo de Polímeros, Facultad de Química y Biología, Universidad de Santiago de Chile, USACH, Casilla 40, Correo 33, Santiago 9170020, Chile; paula.zapata@usach.cl; 5 Grupo de Investigación de Fotoquímica y Fotobiología, Universidad del Atlántico, Carrera 30 No 8-49, Puerto Colombia 081008, Colombia; moises.solano3@hotmail.com

**Keywords:** chitosan beads, red *Tilapia* fillets, *Thymus capitatus*

## Abstract

Red *Tilapia* is one of the most consumed but perishable fish in the world. As a result, it requires preservation methods for safe consumption without affecting its organoleptic characteristics. Chitosan encapsulating essential oils have shown to be an excellent food conservation method. For that reason, we carried out the study of the protective effect on *red Tilapia* fillets with chitosan beads (CB) incorporated with *Thymus capitatus* (TCEO) essential oil at 500, 1000, and 2000 mg/L to assess the conservation of the fillets. The TCEO composition was characterized by gas chromatography-mass spectrometry (CG-MS). For the other side, CB was characterized by Fourier transform infrared spectroscopy (FTIR), X-ray diffraction spectroscopy (XRD), thermogravimetric analysis (TGA), and Scanning electron microscopy (SEM). The protective effect of the beads was tested against the *Gram-positive* and *Gram-negative* bacteria growth for four weeks. The results showed an inhibition effect in *Gram-positive* bacteria at higher TCEO concentration (1000 and 2000 mg/L). Besides that, the pH, total volatile basic nitrogen (T-BNV-N), color, and fillet texture were evaluated as quality attributes. The results suggested that the incorporation of the CB-TCEO allowed a higher contact of the active compounds with the food surface, which reflected more excellent stability. The quality attributes of the fillets were preserved for 26 days, suggesting its uses for the treatment for perishable food.

## 1. Introduction

According to the Food and Agriculture Organization (FAO) [1,2], in 2018, the world fish production reached a total output of 171 million tons per year. Of this amount, 88% was for direct consumption, and 12% was for aquaculture. These statistics demonstrated that fish is one of the most traded food products in the world. However, this product comes along with the most considerable losses. In Colombia, 475 tons of fish are lost annually, equivalent to roughly three million cans of tuna [2].

Fish meat is highly perishable because it undergoes post-mortem physical and biochemical alterations that modify its sensory characteristics. In addition, all its nutrients facilitate microbial growth [3]. During the processing and storage of fish, the deterioration limits the shelf life of the product. For this reason, chemical, sensory, and functional characteristics must be preserved [4].

To prolong food stability, the use of natural preservatives that inhibit the growth of microorganisms has been implemented [5]. Chitosan has been used for the preservation of different food products, and the addition of essential oils has proved to be a good option as a strategy to improve its antimicrobial activity in situ. In addition, chitosan coatings have been widely used in food products because they are adherent, highly biocompatible, transparent, and colorless [6].

One of the most representative characteristics for considering the coatings to be biocompatible as well as edible is that they present low toxicity (with a DL_50_ = 16 mL/kg), at levels similar to sucrose and less than cooking salt [7]. Also, a study of the evaluation of the antibacterial and antioxidant activities of edible chitosan films incorporated with organic essential oils showed that chitosan films containing *Thymus capitatus* were more effective against *Listeria innocua* and *Alcaligenes faecalis* [8]. The authors reported that after the incorporation, the essential oil was trapped in the polymeric matrix and was gradually released to develop an antimicrobial system [9].

Microbial control is an essential parameter for the handling and processing of fish products [10]. Alternatives to preserve foods include the use of polysaccharides coatings, waxes, and carbohydrates to prevent microbial spoilage. Some coatings work synergistically with antimicrobial agents such as extracts and essential oils which delays microbial deterioration, maintaining freshness and color, thereby showing great potential for application in the conservation of food products such as fish fillets [11,12].

For the other side, essential oils such as oregano (*Origanum vulgare*) and thyme (*Thymus vulgaris*), due to their complex mixture of components, have shown excellent activity against a great variety of microorganisms [13]. Besides, if these oils are colloidally protected, they achieve an exciting technology that is used in the food industry, as they prevent food volatilization and extends the fish’s useful life [14].

For the best of our knowledge, it is the first-time using chitosan beads incorporating essential oil of *Thymus capitatus* in *Oreochromis mossambicus* (*red Tilapia*) fillets under refrigeration conditions.

## 2. Experimental

### 2.1. Materials

Fish of the species *Oreochromis mossambicus* (*Red Tilapia*) 25 cm in length were obtained directly from a fish culture for consumption located in the city of Cali, Colombia (Latitude N 3°19′17″, Longitude O 76°31′1831′18″) at 995 m.a.s.l. Each one was gutted, leaving only two portions of fillets, which were placed in resealable polyethylene bags according to the treatments. The experimental units consisted of 50 g fillets with CB with essential oil of TCEO at different concentrations (0, 500, 1000, and 2000 mg/L of TCEO per kg of *Tilapia*). The samples were refrigerated at 4 °C until their use in the different tests during days 1, 5, 9, 13, 16, 19, 23, and 27. All reagents were from Sigma-Aldrich, Palo Alto, CA, USA.

### 2.2. Synthesis

#### Preparation of the CB-TCEO

The beads were prepared from chitosan of medium molecular weight (deacetylation degree 64.17%) and essential oil of *T. capitatus* from Sigma, Palo Alto, CA, USA. The chitosan was dissolved in 1% acetic acid to obtain a final solution of 1.5% (w/v), then, the essential oil was added to obtain the concentrations of the treatments: 500, 1000 and 2000 mg/L and Tween 80 (10% of the essential oil) was added as a surfactant to favor the dispersion of the essential oil. CB-TCEO particles of 6 mm ± 0.15 of diameter were formed by immersion of droplets of 3.5 × 10^−2^ mL of the chitosan solution with essential oil on top, in a 2 N sodium hydroxide solution. The beads obtained were washed with water until neutrality [15]. Encapsulation efficiency was measured using the method reported by Sangsuwan [15]. CB-TCEO where extracted using Soxhlet extraction with THF solvent at 40 °C overnight. Extracts were analyzed by UV/Vis spectrophotometer (Genesys 20 Thermo Scientific, Waltham, MA, USA). Briefly, 0.5 g of CB-TCEO were extracted using 50 mL of THF (99.5%, Applichem Panreac, St. Louis, MO, USA) overnight in a Soxhlet equipment. TCEO dissolved in THF was read at 280 nm (maximum wavelength for carvacrol, the main component of the TCEO) [15]. The percentage of encapsulation efficiency (EE) was calculated using the following equation:

(1) $$\%EE=\frac{Abs\;wash\;TCEO}{Abs\;free\;TCEO}\times 100$$

where *Abs free TCEO* is the absorbance of the TCEO free and *Abs wash TCEO* is the absorbance of the THF used to extract the CB-TCEO.

### 2.3. Characterization

#### 2.3.1. Characterization of the TCEO

Composition analysis of the TCEO was performed by a coupled gas chromatographer Agilent 1200 with an AT 6890 Series plus mass spectrometer (Agilent Technologies, Palo Alto, CA, USA) [16]. TCEO composition was previously reported and Table S1 presents the main components and relative intensities [16].

#### 2.3.2. Characterization of the CB

The beads were dried at 45 °C until reaching a constant weight. The XRD measurements were run on a diffractometer Siemens D5000 (Malvern PANalytical, Jarman Way, Royston, UK) Radiation: k(alpha1) 1.540598 and k(alpha2) 1.544426, from a copper anode with a 45 kV electron accelerator voltage and current to generate 40 mA electrons, with 0.25° and 0.125° incident beam optical grid, 75 mm diffracted beam grid, 0 mm solar grid 0.04 rad and a detector PIXel (Malvern PANalytical, Jarman Way, Royston, UK) in Scanning mode with an active length (°) of 2.5108, step size 0.0197°, range of 2θ from 0–80°, and time per step 304.390 s.

The Thermogravimetric analysis was performed on a Netzsch TG libra 209 instrument (TA instrument, New Castle, DE, USA) adjusted in a working temperature range between 30–700 ± 2 °C.

FTIR analysis was carried out on a Bruker Vector 22 FTIR spectrometer (ThermoFisher Scientific, Waltham, MA, USA), and samples were mixed with analytical grade KBr at 5% w/w. The spectra were recorded in the 4000 to 500 cm^−1^ range, with a resolution of 4 cm^−1^.

SEM analysis was performed on a Scanning electron microscope (JEOL JSM-6490LA, Musashino, Tokyo, Japan) using an acceleration voltage of 20kV, in which the samples were coated with a copper bath.

#### 2.3.3. Antioxidant Activity of the CB-TCEO

Antioxidant activity was measured by the ABTS method reported by Re et al. [17]. To achieve this, radical discoloration was monitored at a wavelength of 734 nm and 25 °C in the ethanolic extract of CB-TCEO (100 mg CB in 3 mL of ethanol for 5 min). After 6 min its absorbance was recorded, and the results were expressed as µmol of Trolox equivalent per gram of sphere and were calculated as the slope ratio of the dose-response curve of the beads and the slope of the dose-response curve of the standard curve.

The antioxidant activity measured by 2,2-diphenyl-1-picrylhydracil (DPPH) method was determined according to Villa-Rodriguez et al. [18] with some modifications. The procedure consisted of dissolving 100 mg of CB in 3 mL of methanol for 5 min. Then, 1 mL of this solution added dropwise to 2 mL of DPPH reagent (dissolved in methanol and adjusted to an absorbance of 0.7 at 517 nm). The tubes were homogenized by vortex agitation for 1 min and stored for two hours in darkness; finally, the absorbance was measured at 517 nm. The results were expressed as µmol of Trolox equivalent per gram of sphere.

#### 2.3.4. Total Phenol Content of the CB-TCEO

The total phenol content was determined using 0.1 g of beads in 3 mL of methanol for 5 min. After that, 0.3 mL of the solution were taken and introduced into the test tubes with 2.5 mL of Folin-Ciocalteu reagent 1 N (Sigma, Milwaukee, WI, USA) and 2 mL of sodium carbonate (20% *w*/*v*). The tubes were mixed and incubated in darkness at 50 °C for 2 h. Finally, the absorbance was measured at 760 nm, and the result was expressed in mg/L of the equivalent of gallic acid, according to the calibration curve.

#### 2.3.5. Bacterial Inhibition In Vitro Capacity of the CB-TCEO

The inhibitory capacity of CB-TCEO was performed with the agar diffusion method with a 24 h growth inoculum of *Bacillus cereus* (ATCC 13061), *Escherichia coli* (ATCC 11775), *Salmonella enterica sub enterica* (ATCC 13311), *Vibrio parahaemolyticus* (ATCC 17802) and *Staphylococcus aureus sub aureus* (ATCC 55804), adjusted to a concentration of 10^6^ CFU/mL, and by adding 100 µL to the Mueller-Hinton agar and spreading it with a sterile handle over the surface of the agar where three CB-TCEO was placed. Incubation was performed at 37 °C for 24 h. Minimum inhibitory concentration (MIC) was defined as the lowest concentration of CB-TCEO that inhibited bacterial growth.

#### 2.3.6. Antibacterial Analysis In Situ in *Tilapia*

For the microbiological analysis of red *Tilapia* in situ, the following Colombian standard methods (NTC) were assessed: total coliform count CFU/g, detection of *E. coli* (NTC 4458, 2007), detection of *Salmonella* spp. (NTC 4574, 2007), detection of *Vibrio cholerae* (Invima FDA: 2004) and *Staphylococcus aureus* count (NTC 4779, 2007).

#### 2.3.7. Total Volatile Nitrogen (TVB-N) Analysis Performed in the *Tilapia* Sample

Total volatile nitrogen in the *red Tilapia* was measured crushing 10 g of the *Tilapia* with 20 mL of distilled water in a conical tube of 50 mL, with the help of Ultraturrax dispersing equipment at 11,000 rpm (IKA-Werke, Staufen, Germany). Subsequently, rinses were made to complete 40 mL, then, 2.0 g of MgO was added in the Kjeldahl UDK 129 distillation column (Velp Scientifica, Crema, Italy). The distillate was collected in 10 mL of boric acid (4%) and then titrated with 0.1 N HCl. The data was expressed as mg N/100 g of the fresh fillet.

#### 2.3.8. pH Analysis in *Tilapia*

For the pH analysis of the *Tilapia*, 20 g of fish muscle were crushed with 20 mL of distilled water in a conical tube, with the Ultraturrax dispersing equipment at 11,000 rpm, and rinses were made to complete 40 mL. Then, the pH values of the *red Tilapia* fillet samples were measured using a pHmeter (Hanna Instruments, Woonsocket, RI, USA) by immersing the electrode in the homogenate [19].

#### 2.3.9. Colorimetric Analysis in *Tilapia*

Color Analysis of the *Tilapia* sample was performed using a CM-600d spectrophotometer (Konica Minolta, Tokyo, Japan) to obtain the color parameters.

#### 2.3.10. Texture Analysis in *Tilapia*

Texture profile analysis (TPA) of the *Tilapia* sample procedure followed Hleap and Velazco reported method [20] with some modifications. Approximately 25 mm thick pieces of *Tilapia* were taken and allowed to stand for 30 min at room temperature before the assay. Using a Universal Tester EZTest EZ-S texturometer (Shimadzu, Kyoto, Japan), a double compression was performed at 75% deformation and at a rate of 2 mm/s with a waiting time of 5 s between compressions, using a 30 mm diameter cylindrical probe. The results analyzed were: hardness (kg/m^2^), elasticity, cohesivity, adhesivity, gomosity, and chewability (kg).

### 2.4. Experimental Design and Statistical Analysis

The study was carried out using a random method in which days of sampling, analysis, and treatments applied to fish fillets were considered. Results were expressed as mean values ± SD, and statistical significance was established at the level of 5% (*p* < 0.05). The Tukey-Kramer test was used to determine differences between treatments for each storage time. All analyses were performed in triplicate.

## 3. Results and Discussion

### 3.1. Characterization of the TCEO

#### Chromatographic Analysis of the TCEO

The encapsulation efficiency of the TCEO in the CB was 80.51 ± 3.6%. This result is similar to those reported in previous studies where the efficiencies of lavender and red thyme essential oils were 63.5 ± 3.2% and 83.9 ± 4.6%, respectively [15]. The retention of essential oils in the CB depends largely on the stability of the forming emulsion [21].

Incorporation of hydrophobic compounds in the hydrophilic polymer favors encapsulation, and the lower moisture content in the polymeric matrix causes a decrease in the plasticity of the beads as water also acts as a plasticizer [18,22].

The main components of the TCEO identified by coupled gas chromatography-mass spectrometry (GC-MS) analysis [16], corresponding to 25 compounds, where 22 are monoterpenes (97.5%), two sesquiterpenes (2.4%), and as a significant component, carvacrol, which is considered to be a potent antibacterial [16].

### 3.2. Characterization of the CB

#### 3.2.1. X-ray Diffraction Analysis of the CB

**Figure 1:**
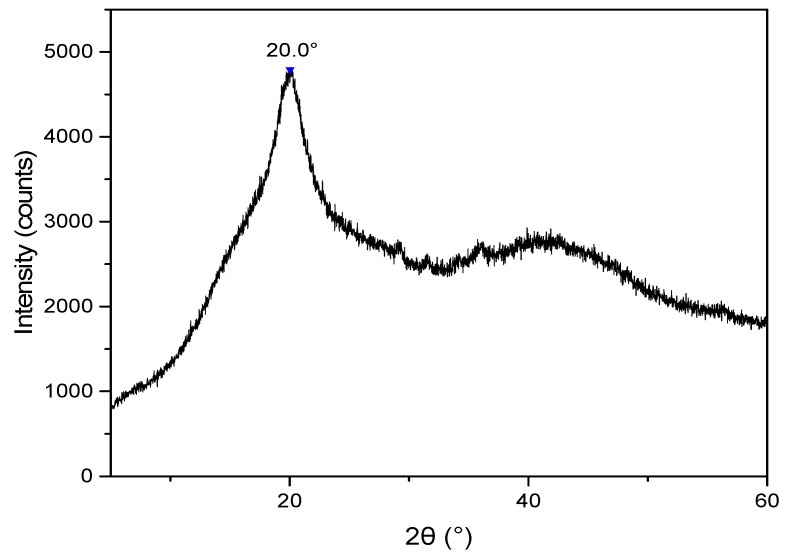
XRD pattern of the chitosan beads (CB).

The XRD (Figure 1) showed the broad central peak of the chitosan at 20° [22], which represents the scattering caused by the allomorphic tendon of the chitosan structure [23]. Also, there is a peak between 10°, but it was gradually absent since there is a good crosslinking in the polymer, reducing the relative crystallinity of the compound and influencing the amorphous properties of the chitosan [23]. On the other hand, the XRD pattern of the CB-TCEO showed a very similar XRD spectra to that of the CB (Figure S1). This could be due to the presence of a very low amount of the TCEO (maximum 2000 ppm), and does not modify the crystallinity of the CB. For this reason, in the CB-TCEO spectrum there is no evidence of an apparent shift or increase in the bands of the XRD [24], particularly the one placed at 20° caused by the allomorphic tendon of the chitosan structure.

#### 3.2.2. Thermogravimetric Analysis of the CB

**Figure 2:**
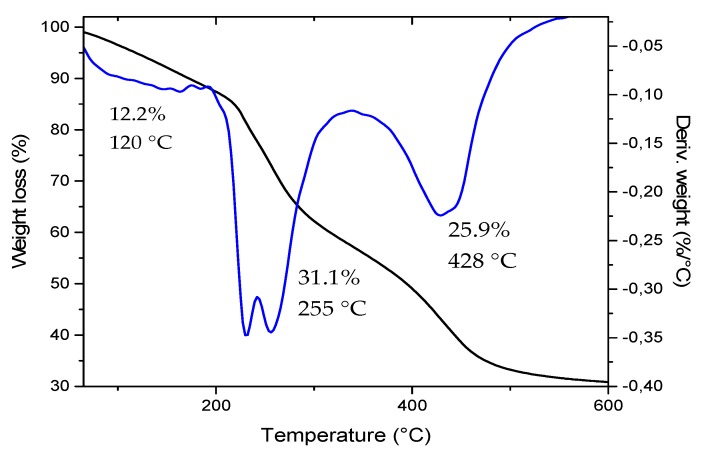
Thermogravimetric analysis of the CB.

The thermogravimetric analysis (TGA) of the chitosan beads (Figure 2) shows the respective mass loss found on chitosan at different temperatures. The first weight loss (12.2%) at 120 °C corresponds to the water molecules trapped in the sample that evaporates when the temperature exceeds the 100 °C [24].

The second most crucial loss occurs at 255 °C, where 31.1% of the total mass is lost, corresponding to the loss of hydrogen bonds between neighboring molecules of the polymeric chitosan chains [25].

Finally, at 428 °C is the third loss of a mass of 25.9% attributed to the degradation and breakage of the backbone of the polymer that generates chains with lower molecular weight [24].

On the other hand, as with the XRD spectra, the degradation temperatures are not affected by the presence of oil in the beads (Figure S2); however, at 120 °C, there is a slow increasing in the mass percentage lost, corresponding to the volatile fraction of the oil (2000 ppm) but maintaining the degradation profile of CB in the other temperatures [26].

### 3.3. Characterization of the Chitosan Beads Incorporating Thymus capitatus Essential Oil (CB-TCEO)

#### 3.3.1. FTIR Analysis

**Figure 3:**
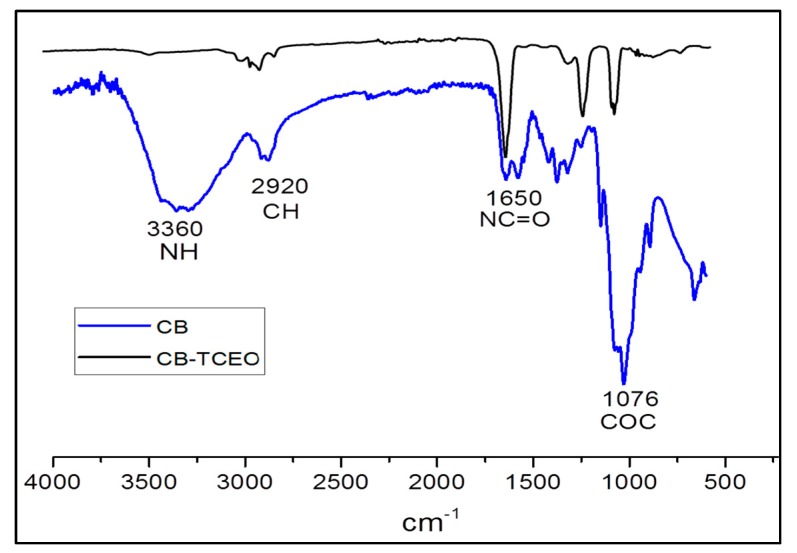
FTIR image of the chitosan beads (CB) and chitosan beads incorporating essential oil (CB-TCEO).

The FTIR characterization of CB and CB-TCEO (Figure 3) shows the fundamental absorption bands corresponding to chitosan [23]. The band corresponding to the amide group C=O appears at 1650 cm^−1^. A substantial, wideband typical of the NH stretch which overlaps the OH band is observed at 3360 cm^−1^. Also, the tension vibration bands of aliphatic –CH at 2920 cm^−1^, and of the C−O−C group of pyranose oxygen at 1076 cm^−1^ are observed. On the other hand, due to the significant number of different groups contributed by the essential oil to the CB-TCEO, the molecular vibration interaction between groups increased, also with the decreasing of the intensity of the fundamental bands. This demonstrates the encapsulation of the oil in CB [25] and the crosslinking of the polymer with the sodium ions, which will decrease the −NH_2_ band intensity.

#### 3.3.2. Antioxidant Activity of CB-TCEO

The antioxidant capacity found in the TCEO relates to the contained phenolic monoterpenes such as carvacrol [27]. Hence, the addition of essential oil in the CB (500, 1000 and 2000 mg/L TCEO) potentiated the antioxidant activity of the measured beads, and for instance, the DPPH showed an increase (from 2085 to 9239 µmol Trolox eq/g CB-TCEO) above the values obtained for pure oil (1833 µmol Trolox eq/g oil) [28]. As another example, the antioxidant activity measured by the ABTS test also showed an increase with the TCEO content but was significantly lower than that for the pure oil.

Finally, the total phenol content (TPC) went from 1.92 to 4.25 mg GAE/g CB-TCEO, which could be responsible for the antioxidant activity observed. These results are presented in Table 1.

#### 3.3.3. Scanning Electron Microscopy (SEM) Analysis of the CB-TCEO

**Figure 4:**
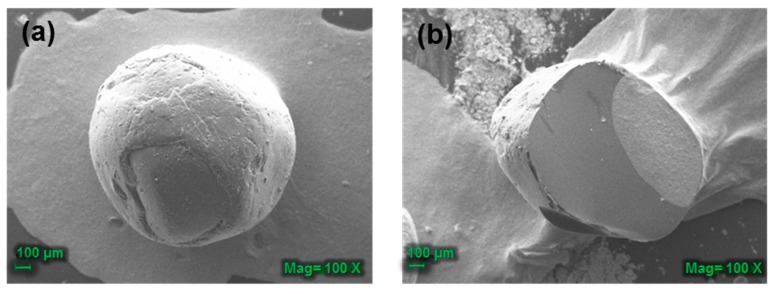
Scanning electron microscopy of (**a**) the surface area of a CB-TCEO and (**b**) the transversal cut area at 100×.

A uniform encapsulation of spherical morphology that is moderately smooth and with small fissures was observed (Figure 4) for the CB-TCEO.

The mechanical resistance of the beads improves with the drying time while the humidity and the evaporation of the essential oil decreases. This mechanical resistance with the drying process gives to the bead’s flexibility, the capacity of resistance to deformation, and high mechanical strength after drying.

However, when the water evaporates, the beads solidify, and the evaporation of the water causes the observed cracks to be generated in the beads [25]. Nevertheless, beads of homogeneous consistency can be observed with no significant defects on their surface. The methodology used allows for beads preparation with efficiency, encapsulating the essential oil without deteriorating and allowing their application in the conservation of fish under refrigeration [29].

#### 3.3.4. Bacteria Inhibition In Vitro Capacity of the CB-TCEO

The CB did not present inhibition zones against the tested strains (Table 2). Usually, chitosan does not diffuse through the agar, and therefore, it only inhibits microorganisms in direct contact with the active sites of chitosan [29].

On the other hand, it was determined that there was a better inhibition of all the bacteria as a function of the increase in the concentration of the essential oil, furthermore, *Gram-positive* bacteria such as *B. cereus* and *S. aureus* were more sensitive than other bacteria at low concentrations of TCEO, similar to the results obtained by Tassou et al. [30]. This indicates that *Gram-positive* bacteria are more sensitive than *Gram-negative* to antimicrobial compounds contained in the essential oil of *Thymus capitatus*. This greater sensitivity is because the cell wall of *Gram-positive* bacteria is thinner than *Gram-negative* bacteria cell wall, allowing penetration to phenolic compounds, aldehydes, ketones, and terpenes, all contained in the essential oil [19]. Finally, chitosan itself has been probed and it was generally recognized that yeasts and molds are the most sensitive group to chitosan, followed by Gram-positive and Gram-negative bacteria [31]. Since, TCEO is very volatile, the antibacterial effect applying the TCEO alone in the fillets will be lost very quickly. However, including the TCEO in CB allows a gradual liberation of the content, preserving, the antibacterial effect and barrier properties over a long period of time, and thereby extending the shelf-life of the tilapia fillets.

However, both strains were inhibited by the CB-TCEO because the essential oil introduces through the lipids of the cell membrane and alter their structure, making them more porous. As a result, there is a leakage of ions and other cellular contents, which can cause cell death. Also, phenolic compounds can change the permeability of the membrane and subsequently, the rate of penetration into the bacterial cell, causing loss of integrity and again, cell death [32].

On the other hand, *Gram-negative* bacteria such as *S. enterica*, *E. coli*, and *V. parehaemoliticus* were less sensitive to CB-TCEO because of their external membrane with a high percentage of lipids. CB-TCEO did not inhibit the growth of *Vibrio parahaemolyticus*.

The results of the antibacterial activity of *T. capitatus* are probably related to the carvacrol content [33], the main compound in the essential oil. This molecule is recognized for its efficiency in inhibiting bacterial growth [34]. Carvacrol causes the disintegration of the external bacterial membrane, followed by the release of lipopolysaccharides, increasing the ATP permeability of the cytoplasmic membrane and the posterior death of the cells [35].

### 3.4. Antibacterial Analysis In Situ in Tilapia

#### 3.4.1. Microbiological Analysis of the Muscle of the Fish Subjected to the Treatments

The microbial study to detect *Salmonella* spp. and *Vibrio cholerae* should be showing the result of their absence in the fish according to the requirements of the standard Colombian (NTC) 1322 method. For that reason, they were inoculated at a concentration of 10 CFU/g in the fillets. After the application of the spheres it was found that CB-TCEO 2000 was effective for inhibiting the growth of the bacteria, accomplishing the requirement of the NTC (Table 3). On the other hand, the effect of the application of CB-TCEO on total Coliforms and *Staphylococcus aureus* coagulase-positive *in vivo* was carried out with spoilage bacteria present in the fish, bacterial counts with results < 10 CFU and < 100 CFU respectively, demonstrating that CB-TCEO 500 and CB-TCEO 2000 were effective in bacterial spoilage control. These results demonstrated that the beads were sufficient to control hazardous bacteria in fillets. They also show that the effectiveness of CB-TCEO against the strains is related not only to the susceptibility to the components of the essential oil but also to parameters such as the initial concentration of the pathogen and the diffusion capacity of the compound within the fish muscle [36].

#### 3.4.2. Determination of Total Volatile Nitrogen (TVB-N)

It is essential to assess the freshness of fish throughout the supply chain to guarantee food quality and safety. TVB-N has been widely considered as a useful indicator of fish freshness over a long period [37]. TVB-N determination is commonly used as an indicator of meat spoilage [38]. This determination is also taken as an index of the quality of fresh or frozen fish because its increase is related to the deterioration by bacteria and the activity of endogenous enzymes [39].

**Figure 5:**
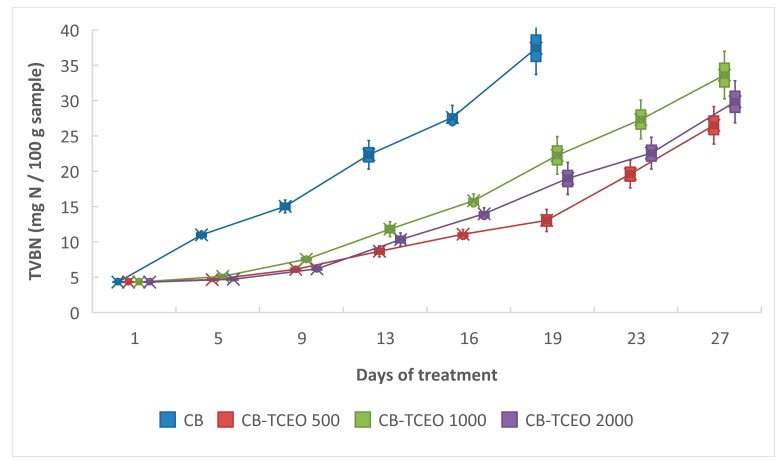
Effect of the CB-TCEO on total volatile basic nitrogen (TVB-N) of *Oreochromis mossambicus* fillet during storage at 4 °C.

The initial value of TVB-N was 4.39 mg of N/100 g of fresh sample (Figure 5). This value showed a variable behavior during the storage period. The results obtained showed an inhibitory effect of CB-TCEO in the formation of TVB-N, possibly related to the inhibition of the microorganism’s growth in the fillets.

The values of TVB-N remained below the consumption limit (25–35 mg/100 g) during 19 days for all the fillets with the CB-TCEO, but not for the control. Wang et al. [40] reported a decrease of the TVB-N value under the limit allowed during 14 days with the addition of essential oil of garlic and chitosan films [40]. In our results, as the storage time increased, there was a continuous increase in the value of TVB-N in all samples, but with significant variation. After 27 days, values of 26.5, 33.61, and 29.84 mg N/100 g were obtained for treatments with 500, 1000, and 2000 mg/L of TCEO, respectively. These values were much lower than those of the fish without the beads, which on day 19 exceeded 35 mg N/100 g, indicating a higher degree of freshness in the fillets with the treatments.

CB-TCEO 500 could have performed better because an excess of essential oil may have decreased the crystallinity of the chitosan, preventing the effective retention of the oil, thus losing its efficiency [41].

According to the FAO [2], the consumption limit of TVB-N is 25–35 mg/100 g. Based on this, fish remained consumable when refrigerated at 4 °C until 13 days without treatment, 21 days with 1000 mg/L, 23 days with 2000 mg/L and up to 26 days with 500 mg/L of essential oil. These results show that treatment with CB-TCEO effectively increases the stability of the fish under storage conditions.

#### 3.4.3. pH Analysis of the *Tilapia* Muscle

The initial pH in the fish muscle can vary immediately after death. This variation depends on the form of capture, season, species, diet, and stress severity. The initial pH value, in this case, was 6.51 and increased significantly *(p* < 0.05) in all treatments (Table 4). During storage, it was observed that the pH value increased less with a higher concentration of the oil in the beads.

A significant increase of pH in fish muscle indicates an accumulation of alkaline compounds, mainly ammonia and trimethylamine (TMA), which are leading derivatives of microbial activity. This indicates that higher concentrations of essential oil reduce the microbial activity in the fish.

#### 3.4.4. Colorimetric Analysis of the *Tilapia* Sample

**Figure 6:**
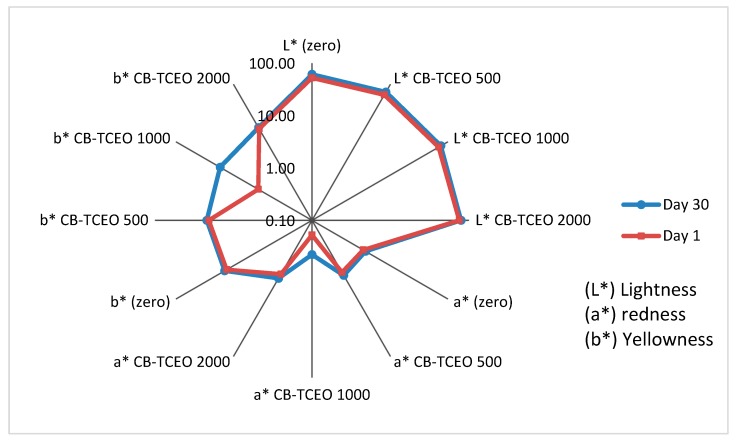
Effect of CB-TCEO on the color parameters of the *Tilapia* sample. L* = Lightness; a* = redness; b* = yellowness (CIELab, color coordinates).

The color coordinates of the *Tilapias* are shown in Figure 6. Lightness (L*) was significantly affected by the addition of CB-TCEO. Lightness is related to factors such as concentration, type of pigments, and water content [42]. Lightness increased with essential oil content because the loss of water from the fish muscle increased the concentration of solutes outdoors. Also, the interaction of the main components of the essential oil (carvacrol) with proteins causes their splitting [32].

On the other hand, for the red-green coordinate, redness (a*) did not show significant differences with respect to the control, but the days of storage. In this case, redness increased, indicating that this parameter is influenced by the storage time and not by the treatments. Also, this process occurs possibly due to the precipitation of myoglobin [42].

For the yellow-green coordinate, yellowness (b∗) results showed significant differences concerning control, treatments, and storage time. Yellowness increased when the concentration of essential oil increased, possibly due to protein splitting [43].

**Figure 7:**
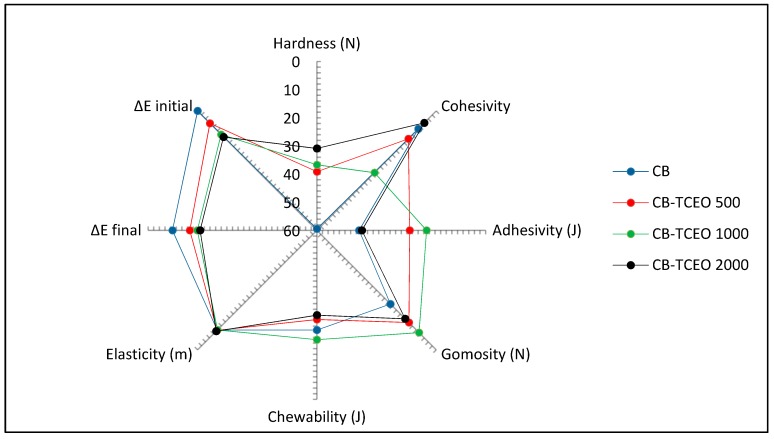
Texture Profile Analysis of the *Tilapia* sample.

Finally, delta E parameter (ΔE) (Figure 7) shows that the difference in color was significantly different (*p* < 0.05) in all treatments, indicating an impact of the CB-TCEO in the fillet’s color.

#### 3.4.5. Texture Profile Analysis (TPA) of the *Tilapia* Sample

Six texture parameters (Figure 7) were measured from the typical curve of a texture profile analysis (TPA) for the *Tilapia* fillets.

Loss of texture is due to the natural deterioration of proteins, caused by the loss of water in the muscle as well as the degradation by microbial action [43]—another factor associated with the deterioration in the water retention capacity. When a fish fillet is fresh, its retention capacity is very high and decreases as the deterioration advances [44]. It should be noted that the elasticity varied little between treatments and variables such as chewability and gomosity showed a slight tendency to increase as storage time increased in the control samples with a higher concentration of TCEO. Adhesivity also gave positive values, indicating that the texture of the *Tilapia* muscle is sticky or adherent to the palate, which leads to necessary work to remove it [20].

In contrast, the hardness showed a significant decrease in the control and more for the CB-TCEO 500 samples during the storage time.

The highest value for the hardness parameter was 65.5 N and was obtained for the sample with CB on day 19 as a cause of the decrease in moisture retention capacity leading to the hardening. In contrast, the lowest value was obtained on day 27 for the sample with CB-TCEO 2000, indicating a smaller loss of moisture in the fillets that contains the more significant concentration of TCEO.

These results agree with those exposed by Rahman (2005) [45], where they demonstrated that hardness and chewability increase exponentially with decreasing moisture content, while adhesivity, cohesivity, and elasticity increase exponentially with the decreasing of the moisture content.

## 4. Conclusions

The morphology of the beads and FTIR analysis showed that the incorporation of the essential oil is carried out homogeneously without affecting the morphology of the beads, which favors the gradual release of the essential oil as the chitosan degrades naturally.

The CG-MS analysis of the TCEO showed that carvacrol was the main component and it contributed a strong antibacterial effect in the fish. For that reason, the addition of CB-TCEO decreases the rate of deterioration of *Oreochromis mossambicus* in refrigerated conditions at 4 °C by the microbiological and the TVB-N parameters of the fish. The results of the analysis in situ for the fillets demonstrated that they remained fresh for a more extended period, with an increase of 13 to 21 days along with the concentration of essential oil incorporated. This increase is mainly due to the inhibition of the activity of microorganisms such as total coliforms, *S. aureus*, and microbes associated with fish deterioration such as *Vibrio parahaemolyticus* and *Salmonella enterica sub enterica,* which were demonstrated to be inhibited under in vitro conditions by CB-TCEO. Additionally, the addition of the beads contributed to the increase of the antioxidant capacity in the fillets, increasing the protection against possible oxidations that can also deteriorate the quality of the product.

Finally, the color and texture parameters, such as hardness, demonstrated that CB-TCEO treatment to the fillets improves stability up to 26 days, making them safe for human consumption.

## Figures and Tables

**Table 1:** Antioxidant activity of the TCEO, CB, and CB-TCEO samples.

Sample	DPPH µmol Trolox (eq/g TCEO)	ABTS µmol Trolox (eq/g TCEO)	TPC (mg GAE/g TCEO)
TCEO	1833 ± 32 ^a^	12.50 ± 0.008 ^e^	0.32 ± 0.03 ^a^
CB	2085 ± 27 ^b^	6.89 ± 0.006 ^a^	1.92 ± 0.05 ^b^
CB-TCEO 500	6200 ± 23 ^c^	7.53 ± 0.011 ^b^	3.36 ± 0.06 ^c^
CB-TCEO 1000	7624 ± 21 ^d^	8.11 ± 0.06 ^c^	3.51 ± 0.06 ^d^
CB-TCEO 2000	9239 ± 20 ^e^	8.95 ± 0.05 ^d^	4.25 ± 0.07 ^e^

Different superscript letter in a column means significant difference (*p* < 0.05). The ± values are the standard deviation (±SD) with *n* = 3.

**Table 2:** Inhibitory activity of incorporated CB with different concentrations of the TCEO.

Sample	The Diameter of the Inhibition Zone (mm)				
	*B. cereus*	*S. aureus*	*S. enterica*	*E. coli*	*V. parahaemolyticus*
CB	-	-	-	-	-
CB-TCEO 500	9.4 ± 1.5 ^a^	5.7 ± 1.0 ^a^	4.2 ± 0.8 ^a^	2.0 ± 0.5 ^a^	-
CB-TCEO 1000	22.9 ± 1.9 ^b^	19.8 ± 2.1 ^b^	17.5 ± 2.5 ^b^	15.3 ± 1.9 ^b^	13.1 ± 1.6 ^a^
CB-TCEO 2000	37.2 ± 2.5 ^c^	34.1 ± 2.3 ^c^	30.8 ± 3.9 ^c^	25.6 ± 2.5 ^c^	23.5 ± 2.7 ^b^
Chloramphenicol 250 mgL^−1^	40.0 ± 1.6 ^c^	39.5 ± 2.0 ^d^	39.7 ± 2.1 ^d^	37.2 ± 1.8 ^d^	36.1 ± 3.2 ^c^

(-) Total increase; In a column, a different superscript letter (a–d) means significant difference (*p* < 0.05); The ± values are the standard deviation (±SD) with *n* = 3.

**Table 3:** Microbiological parameters of *Oreochromis mossambicus* fillets refrigerated at 4°C according to NTC 1322.

Day	CB-TCEO	Microorganism (UFC/g)			
		*Salmonella* spp. *	Vibrio cholerae *	Coliforms Yotal	*S. aureus* Coagulase-Positive
	Requirement NTC 1322	Absence	Absence	<10	<100
1	**0**	10	10	<10	<100
	500	<10	<10	Absence	<10
	1000	<10	<10	Absence	<10
	2000	Absence	Absence	Absence	Absence
5	**0**	<100	<100	<10	<100
	500	<10	<10	Absence	<10
	1000	<10	<10	Absence	<10
	2000	Absence	Absence	Absence	Absence
9	**0**	<100	<100	<100	<1000
	500	<10	<10	Absence	<100
	1000	<10	<10	Absence	<10
	2000	Absence	Absence	Absence	Absence
13	**0**	<1000	<1000	<100	<1000
	500	<100	<100	Absence	<100
	1000	<10	<10	Absence	<10
	2000	Absence	Absence	Absence	Absence
16	**0**	<1000	<1000	<1000	<10,000
	500	<100	<100	Absence	<100
	1000	<10	<10	Absence	<10
	2000	Absence	Absence	Absence	Absence
19	**0**	<10,000	<10,000	<1000	<10,000
	500	<100	<100	Absence	<100
	1000	<100	<100	Absence	<10
	2000	Absence	Absence	Absence	Absence
23	**0**	<10,000	<10,000	<1000	<10,000
	500	<100	<100	Absence	<1000
	1000	<100	<100	Absence	<100
	2000	Absence	Absence	Absence	Absence
27	**0**	<10,000	<10,000	<1000	<100,000
	500	<1000	<1000	Absence	<1000
	1000	<100	<100	Absence	<100
	2000	Absence	Absence	Absence	Absence

* inoculated at a concentration of 10 CFU/g.

**Table 4:** Effect of the application of CB-TCEO on the pH in the muscle of *Oreochromis mossambicus*.

Sample	pH/Day							
	1	5	9	13	16	19	23	27
CB	6.51 ± 0.01 ^a^	6.58 ± 0.02 ^c^	6.61 ± 0.03 ^b^	6.65 ± 0.02 ^c^	6.72 ± 0.05 ^c^	6.80 ± 0.02 ^c^	6.85 ± 0.03 ^c^	6.90 ± 0.03 ^c^
CB-TCEO 500	6.51 ± 0.01 ^a^	6.53 ± 0.03 ^b^	6.57 ± 0.04 ^b^	6.60 ± 0.03 ^b^	6.64 ± 0.02 ^b^	6.71 ± 0.01 ^b^	6.76 ± 0.03 ^b^	6.81 ± 0.02 ^b^
CB-TCEO 1000	6.51 ± 0.02 ^a^	6.47 ± 0.03 ^a^	6.51 ± 0.03 ^a^	6.52 ± 0.02 ^a^	6.58 ± 0.03 ^a^	6.63 ± 0.03 ^a^	6.68 ± 0.01 ^a^	6.73 ± 0.03 ^a^
CB-TCEO 2000	6.50 ± 0.02 ^a^	6.44 ± 0.04 ^a^	6.47 ± 0.04 ^a^	6.50 ± 0.03 ^a^	6.55 ± 0.05 ^a^	6.60 ± 0.02 ^a^	6.65 ± 0.04 ^a^	6.70 ± 0.01 ^a^

In a column, a different superscript letter (a–c) means significant difference (*p* < 0.05), the ± values are the standard deviation (±SD) with *n* = 3.

## Supplementary Materials

The following are available online at https://www.mdpi.com/2218-273X/9/9/458/s1. Figure S1: XRD pattern of the chitosan beads with *Thymus capitatus* essential oil (CB-TCEO); Figure S2: Thermogravimetric analysis of the chitosan beads with *Thymus capitatus* essential oil (CB-TCEO); Table S1: Volatile compounds expressed as area percentage, identified in the *Thymus capitatus* essential oil.
