# Supplementary material for: Chitosan Beads Incorporated with Essential Oil of Thymus capitatus: Stability Studies on Red Tilapia Fillets
Source: Biomolecules. 2019 Sep 7;9(9):458. doi: 10.3390/biom9090458 (PMC6769681; doi:10.3390/biom9090458)
Supplement: Supplementary file 1 [file biomolecules-09-00458-s001.pdf]

biomolecules

Article

Chitosan beads incorporated with essential oil of *Thymus capitatus*: stability studies on red *Tilapia* fillets

Mayra Alejandra Valencia Junca[1], Cesar Valencia[2], Edwin Flórez López [1], Johannes Delgado Ospina[3], Paula A. Zapata[4], Moisés Solano[5], and Carlos David Grande Tovar [5,*]

[1] Grupo de investigación en química y biotecnología QUIBIO, Universidad Santiago de Cali, Calle 5 No 62-00, 760035 Cali, Colombia; aleja_valencia2201@hotmail.com (M.V.J.); edwin.florez00@usc.edu.co (E.F.L.)
[2] SIMERQO Laboratorio de Polímeros, departmento de química, Universidad del Valle, Calle 13 No 100-00, 760031, Cali, Colombia; cesar.valencia@correounivalle.edu.co (C.V.)
[3] Grupo de Investigación Biotecnología, Facultad de Ingeniería, Universidad de San Buenaventura Cali, Carrera 122 # 6-65, 76001 Cali, Colombia; jdelgado1@usbcali.edu.co (J.D.)
[4] Grupo de Polímeros, Facultad de Química y Biología, Universidad de Santiago de Chile, USACH, Casilla 40, Correo 33, Santiago, Chile; paula.zapata@usach.cl (P.Z.)
[5] Grupo de investigación de fotoquímica y fotobiología, Universidad del Atlántico, Carrera 30 No 8-49, 081008, Puerto Colombia, Colombia (C.D.G.T.)
* Correspondence: carlosgrande@mail.uniatlantico.edu.co

Received: date; Accepted: date; Published: date

1. Supporting figures

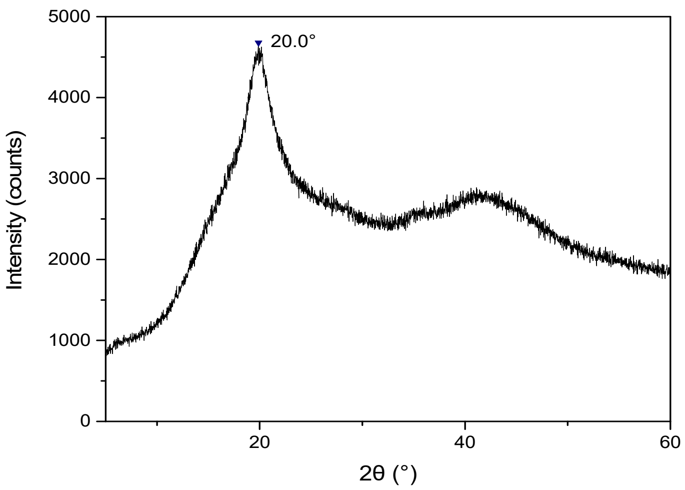

Figure S1. XRD pattern of the chitosan beads with *Thymus capitatus* essential oil (CB-TCEO)

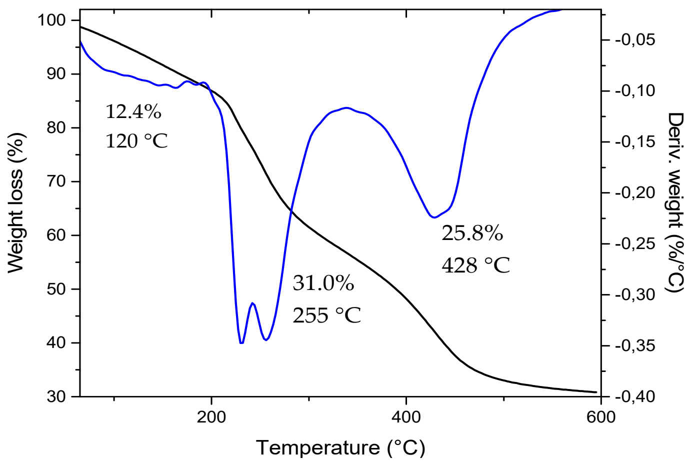

Figure S2. Thermogravimetric analysis of the chitosan beads with *Thymus capitatus* essential oil (CB-TCEO)

Table S1. Volatile compounds expressed as area percentage, identified in the *Thymus capitatus* essential oil [1].

	Compound	RT	Amount Relative (%)	*KI
Monoterpenes hydrocarbons	Tricyclene	17.15	<0.1	920
	α-Thujene	17.26	0.1	923
	α-Pinene	17.65	1.5	935
	α-Fenchene	18.43	0.3	951
	β-Pinene	19.67	0.3	981
	β-Myrcene	20.03	2.0	991
	p-Mentha-1(7),8-diene	20.78	0.1	992
	α-Phellandrene	20.88	0.2	1005
	δ–3–Carene	20.99	<0.1	1012
	1,4-Cineole	21.23	<0.1	1014
	α-Terpinene	21.34	1.5	1018
	p-Cymene	21.74	13.2	1026
	Limonene	21.89	0.4	1033
	1,8-cineole	22.05	0.4	1033
	γ-Terpinene	23.13	8.7	1064
	N.I. (M+154)	23.63	0.1	
Monoterpenes oxygenated	Terpinolene	24.24	0.2	1078
	Linalool	24.73	1.9	1100
	Borneol	27.87	0.3	1165
	Terpinen-4-ol	28.15	0.6	1190
	α-Terpineol	28.79	0.1	1200
	Thymol	32.07	6.4	1266
	Carvacrol	32.63	59.3	1278
	trans-β-Caryophellene	37.34	2.2	1424
Sesquiterpenes oxygenated	Caryophyllene oxide	42.50	0.2	1581

*KI is the Kovatz Index relative to C5–C24 n-alkanes on the DB-5 column

1. Grande-Tovar, C.D.; Serio, A.; Delgado-Ospina, J.; Paparella, A.; Rossi, C.; Chaves-López, C. Chitosan films incorporated with Thymus capitatus essential oil: mechanical properties and antimicrobial activity against degradative bacterial species isolated from tuna (Thunnus sp.) and swordfish (Xiphias

gladius). *J. Food Sci. Technol.* **2018**, *55*, 4256–4265, doi:10.1007/s13197-018-3364-y.
